# Treatment patterns of systemic drug use in Japanese patients with plaque psoriasis: A retrospective chart review

**DOI:** 10.1111/1346-8138.17038

**Published:** 2023-11-30

**Authors:** Yayoi Tada, Mayumi Komine, Yukari Okubo, Katsuyoshi Habiro, Katsuki Tsuritani, Akimichi Morita

**Affiliations:** ^1^ Department of Dermatology Teikyo University Tokyo Japan; ^2^ Department of Dermatology Jichi Medical University Tochigi Japan; ^3^ Department of Dermatology Tokyo Medical University Tokyo Japan; ^4^ Tyk2 and Immunology Medical, Bristol Myers Squibb K.K. Tokyo Japan; ^5^ Department of Geriatric and Environmental Dermatology Nagoya City University Graduate School of Medical Sciences Nagoya Japan

**Keywords:** biologics, PDE4 inhibitor, psoriasis, systemic drug, treatment pattern

## Abstract

Plaque psoriasis (PsO) is a chronic immune‐mediated inflammatory disease with skin lesions accompanied by an inflammation‐related comorbidity risk. The development of various oral drugs and biologics for PsO has provided increasing systemic treatment options for patients with PsO, and the guidance regarding the use of biologics and PsO treatment schemes are widespread in Japan. However, no comprehensive guidelines regarding systemic drug use are available, and the current treatment patterns of systemic drugs for PsO in Japan remain unclear. We conducted a retrospective chart review to clarify the current treatment patterns of systemic drugs for PsO. We enrolled 114 patients who started systemic drugs for PsO between January 2017 and December 2020 at four institutes, with a mean follow‐up of 37.2 months. The mean disease duration was 7.8 (standard deviation 9.5) years at the systemic drug initiation. Of all the patients, 78.1% started with oral drugs (phosphodiesterase [PDE] 4 inhibitors 56.1%. calcineurin inhibitors 14.0%. vitamin A derivatives 7.9%), whereas 21.9% started with biologics (interleukin [IL]‐17 inhibitors 9.6%. tumor necrosis factor inhibitors 7.0%. IL‐23 inhibitors 3.5%. IL‐12/23 inhibitors 1.8%). Oral drugs had shorter drug persistence than biologics: the 12‐month persistence of the oral drugs vitamin A derivative, calcineurin inhibitor, and PDE4 inhibitor, was 35.5%, 25.8%, and 60.1%, respectively, compared with that of the biologics IL‐23 and IL‐17 inhibitors, which was 85.6% and 84.7%, respectively. During the study period, the incidence of treatment changes was 59.1/100 patient‐years. Lack of efficacy was the most common reason for treatment changes from monotherapy (34.1%). This retrospective medical chart review allowed us to understand the real‐world, long‐term treatment patterns of systemic drugs for PsO and the relationships between the reasons for treatment changes and subsequent treatment selection, indicating that there is still room for improvement in the appropriate use of systemic drugs for PsO in Japan.

## 1 INTRODUCTION

Plaque psoriasis (PsO) is a chronic systemic immune‐mediated inflammatory disease that predominantly affects the skin and manifests with various morphologies and wide distribution.^1^ Recent epidemiologic studies have estimated that the prevalence of PsO is 0.34% in Japan.^2^ Patients with lesions on visible body parts have decreased quality of life, leading to psychological stress and negatively affecting work productivity.^3^, ^4^

It is unclear whether PsO triggers multiple comorbidities or vice versa, but chronic systemic inflammation negatively affects PsO severity and increases cardiovascular risk. Aside from lesions on the body, PsO is associated with multiple comorbidities, such as atherosclerosis and cardiovascular disease,^5^ including myocardial infarction and stroke,^6^, ^7^ metabolic syndrome, hypertension, type 2 diabetes mellitus, and dyslipidemia.^8^, ^9^ Some evidence suggests that tumor necrosis factor (TNF) inhibitors and interleukin (IL)‐17 inhibitors reduce cardiovascular risk in patients with PsO.^10^ More importantly, it has been reported that approximately 20% of patients with PsO develop psoriatic arthritis with irreversible joint destruction.^11^ Reportedly, patients receiving IL‐12/23 or IL‐23 inhibitors have a lower risk of developing inflammatory arthritis than those prescribed TNF inhibitors,^12^ therefore appropriate long‐term treatments for PsO are required to control systemic inflammation.

As of December 2020, four types of oral drugs, including phosphodiesterase 4 (PDE4) inhibitors, antifolates, calcineurin inhibitors, vitamin A derivatives, and 11 biologics, had been authorized in Japan as systemic drugs for PsO. These systemic drugs alleviated PsO severity and improved the patients' quality of life.^13^, ^14^, ^15^ The treatment scheme for Japanese patients with PsO based on the PsO treatment pyramid plan^16^ has reached a consensus, and the Japanese Dermatological Association (JDA) has released guidance regarding the use of biologics and Janus kinase inhibitors for PsO^17^, ^18^; however, there are no comprehensive guidelines regarding systemic drug use for PsO.^13^, ^14^ The selection of systemic drugs for each patient among various types of oral drugs or biologics therefore depends on the physician's judgment and patient preference. In addition, the long‐term treatment courses for PsO can vary among Japanese patients. As patients with PsO are likely to undergo lifelong treatment, the treatment selection for each patient can also affect healthcare costs.

Retrospective studies on systemic drugs for PsO were conducted in the USA and Japan using claims databases collected from healthcare insurance data to clarify treatment patterns (i.e., the proportion of systemic drugs selected, adherence, and drug discontinuation) during the 12‐month follow‐up period in patients with PsO.^19^, ^20^, ^21^ PsO is a chronic disease requiring long‐term treatment for several years. Therefore, further investigations to clarify the longer‐term systemic treatment courses and outcomes for PsO are needed to continuously provide appropriate systemic drugs for patients with PsO who may require lifelong treatment. Furthermore, systemic treatment options for PsO are expanding. After this study period, a TNF‐α inhibitor, an IL‐17 inhibitor, and a tyrosine kinase 2 inhibitor were approved in Japan.^14^, ^22^, ^23^ Therefore, understanding the correct administration of drugs tailored to each patient's needs is increasingly crucial.

This study aimed to investigate the long‐term treatment course, patterns of treatment change for each systemic drug (e.g., drug interruption and switching to other treatments), reasons for treatment change, and the relationship between the reasons for treatment change and subsequent treatment by analyzing the medical records of patients with PsO for at least 2 years.

## 2 METHODS

### 2.1 Study design and data sources

This multicenter, non‐interventional, retrospective observational study was conducted at four JDA‐certified medical institutions in Japan between January 1, 2017, and December 31, 2020. All four institutes were tertiary medical institutions authorized to dispense biologics to patients with PsO. We aimed to collect data from 120 patients, based on existing research.^24^ Data of patients with PsO were collected from medical records using electronic data capture systems. All patients who started a systemic drug for PsO on or after January 1, 2017, at the four tertiary medical institutions and who met the eligibility criteria were enrolled in this study. Treatment courses with systemic drugs for PsO and the reasons for treatment changes, including switching to other systemic drugs, addition of other drugs, and treatment interruption, in clinical settings were collected retrospectively. All data were anonymized prior to analysis. The ethical review boards of the institutions involved in this study approved the study protocol and waived the requirement for informed consent because the study design was non‐experimental and retrospective. This study was conducted in accordance with the International Society for Pharmacoepidemiology Guidelines for Good Pharmacoepidemiology Practices and applicable regulatory requirements. This study was registered at ClinicalTrials.gov under the identification number NCT04826536.

### 2.2 Inclusion and exclusion criteria

Eligible patients were defined as those who were aged ≥20 years and had a diagnosis of PsO at the start of follow‐up, had initiated a systemic drug such as TNF‐α inhibitors (infliximab, adalimumab, and certolizumab pegol), IL‐12/23 inhibitors (ustekinumab), IL‐23 inhibitors (guselkumab, risankizumab, and tildrakizumab), IL‐17 inhibitors (secukinumab, ixekizumab, and brodalumab), PDE4 inhibitors (apremilast), antifolates (methotrexate), calcineurin inhibitors (cyclosporine), or vitamin A derivatives (etretinate) during the study period and had been followed up for at least 2 years after the initiation of systemic treatment. Patients were excluded if they participated in another clinical trial for PsO before or during the study period, if they had psoriatic arthritis, guttate psoriasis, erythrodermic psoriasis, or pustular psoriasis at the start of follow‐up, and if they had already received any systemic drugs investigated in this study before the diagnosis of PsO.

### 2.3 Data collection, definition of treatment change, and other variables of interest

Demographics and patient background characteristics, including age, sex, duration of PsO, and comorbidities, were collected for all patients at the start of the follow‐up. Based on the medical records at the time of each hospital visit, the treatment courses of systemic drugs for PsO were collected during the study period, including prescribed drug names and their categories, and reasons for treatment changes, to clarify the persistence of systemic drug use and patterns of treatment changes. In addition, data on concomitant phototherapy (ultraviolet B [UVB], psoralen ultraviolet A [PUVA]) was also collected. Treatment changes were categorized into six types: switching to another systemic drug, adding another drug, reducing a drug from multidrug therapy, treatment interruption, starting systemic drug after interruption, and restarting the previous systemic drug after interruption. Switching to another drug, adding another drug, and reducing a drug from multidrug therapy were counted as a case if the treatment change occurred on the visit day scheduled for a prescription. The interruption was counted as a case if the patient did not receive the prescription on the visit day scheduled for the prescription or if the patient did not visit the medical institution for the prescription within 28 days after the scheduled prescription date. Additionally, the number of interruptions and the reasons were counted by the definition of the interruption period of ≥28, ≥60, ≥90, and ≥ 180 days. The treatment duration was defined from the start of systemic drug or treatment change to the day immediately before the next treatment change or to the end of follow‐up. The reasons for treatment changes were categorized into five types: occurrence of a new type of PsO (e.g., psoriatic arthritis, guttate psoriasis, erythrodermic psoriasis, and pustular psoriasis), adverse events (AEs), lack of efficacy, sufficient efficacy, and others. All data regarding treatment changes and the reasons for treatment change were linked to the systemic drugs prescribed until the time of treatment change. Psoriasis Area and Severity Index (PASI) scores were calculated to measure the severity and extent of the PsO lesions.^25^ The frequency of treatment change per 100 patient‐years was calculated as the number of treatment changes divided by the corresponding patient‐years of observation.

### 2.4 Statistical analysis

Patient characteristics for each systemic drug started during this study were analyzed using descriptive statistics (mean, standard deviation [SD]). Frequencies and proportions (%) were calculated for categorical or ordinal variables. The Kaplan–Meier method was used to estimate the persistence of initial systemic drugs during the study and all systemic drugs during the study period based on the number of events and censored data. Statistical analyses were performed using SAS software version 9.4 (SAS Institute Inc., Cary, NC, USA).

## 3 RESULTS

### 3.1 Study population and the initial systemic drug

The study population consisted of 114 patients, 69.3% were men, and the mean (SD) age was 52.6 (16.3) years (Table 1). The mean (SD) duration from the first diagnosis for PsO to the start of systemic drug use was 7.8 (9.5) years, and systemic treatment courses were followed up for a mean period of 37.2 months. Among the study population, 78.1% started with oral drugs (PDE4 inhibitors 56.1%, calcineurin inhibitors 14.0%, vitamin A derivatives 7.9%) as the initial systemic drug, whereas the remaining 21.9% started with biologics (IL‐17 inhibitors 9.6%, TNF inhibitors 7.0%, IL‐23 inhibitors 3.5%, IL‐12/23 inhibitors 1.8%) (Table 1). All patient populations for each initial systemic drug had comparable PsO durations and follow‐up periods. The mean (SD) PASI score at baseline was 12.7 (8.9), and 51.4% (19/37) of patients had PASI ≥10. Patients who had started TNF‐inhibitors, IL‐17 inhibitors, or vitamin A derivatives during the study tended to have a higher mean PASI score (18.7, 18.5, and 18.8, respectively), whereas those who had received PDE4 inhibitors or calcineurin inhibitors tended to have a lower mean PASI score (11.0 and 6.9, respectively). Comorbidities were observed in 69.3% of the study population, and the most common comorbidity was dyslipidemia (20.2%), followed by hypertension (19.3%) and diabetes mellitus (15.8%). There was no clear trend between comorbidities and the initial systemic drugs started during the study (Supporting Information Table S1).

**TABLE 1:** Patients background characteristics and initial drug type.

Background characteristics	All	Initial drug started during the study period							
		Biologics (all)	Biologics				Oral drugs		
			TNFi	IL‐12/23i	IL‐23i	IL‐17i	PDE4i	CaNi	Vit A deriv
Patients, *n* (%)	114 (100)	25 (21.9)	8 (7.0)	2 (1.8)	4 (3.5)	11 (9.6)	64 (56.1)	16 (14.0)	9 (7.9)
Sex (male), *n* (%)	79 (69.3)	17 (68.0)	4 (50.0)	2 (100.0)	2 (50.0)	9 (81.8)	45 (70.3)	11 (68.8)	6 (66.7)
Age^a^ (years), mean (SD)	52.6 (16.3)	44.1 (14.9)	43.0 (13.0)	71.0 (14.1)	38.3 (13.5)	42.2 (13.3)	56.9 (14.5)	44.6 (15.0)	59.8 (21.0)
Duration of psoriasis^a^ ^,^ ^b^ (years), mean (SD)	7.8 (9.5)	8.4 (8.7)	9.4 (11.2)	8.0 (4.2)	7.3 (9.9)	8.1 (7.9)	7.9 (9.7)	7.4 (10.6)	6.6 (9.7)
Follow‐up period (months), mean (SD)	37.2 (6.2)	37.1 (6.3)	38.2 (5.7)	44.4 (0.3)	27.6 (2.4)	38.5 (4.7)	37.2 (6.1)	37.8 (6.0)	36.6 (7.8)
PASI score, *n*	37	13	4	0	3	6	16	5	3
PASI score^c^, mean (SD)	12.7 (8.9)	15.5 (8.7)	18.7 (5.7)	– (−)	5.3 (0.7)	18.5 (8.9)	11.0 (9.8)	6.9 (3.4)	18.8 (5.7)
PASI <10	18 (48.6)	4 (30.8)	0 (0.0)	0 (−)	3 (100.0)	1 (16.7)	10 (62.5)	4 (80.0)	0 (0.0)
PASI ≥10	19 (51.4)	9 (69.2)	4 (100.0)	0 (−)	0 (0.0)	5 (83.3)	6 (37.5)	1 (20.0)	3 (100.0)
Comorbidity, yes, *n* (%)	79 (69.3)	15 (60.0)	6 (75.0)	2 (100.0)	1 (25.0)	6 (54.5)	48 (75.0)	7 (43.8)	9 (100.0)
Dyslipidemia^d^	23 (20.2)	3 (12.0)	1 (12.5)	0 (0.0)	1 (25.0)	1 (9.1)	16 (25.0)	1 (6.3)	3 (33.3)
Hypertension^d^	22 (19.3)	4 (16.0)	2 (25.0)	1 (50.0)	0 (0.0)	1 (9.1)	15 (23.4)	0 (0.0)	3 (33.3)
Diabetes mellitus^d^	18 (15.8)	3 (12.0)	2 (25.0)	0 (0.0)	0 (0.0)	1 (9.1)	12 (18.8)	1 (6.3)	2 (22.2)
Moderate liver disease^d^	12 (10.5)	3 (12.0)	1 (12.5)	0 (0.0)	1 (25.0)	1 (9.1)	8 (12.5)	1 (6.3)	0 (0.0)
Hay fever^d^	9 (7.9)	1 (4.0)	0 (0.0)	0 (0.0)	0 (0.0)	1 (9.1)	3 (4.7)	3 (18.8)	2 (22.2)
Hyperuricemia^d^	8 (7.0)	3 (12.0)	1 (12.5)	1 (50.0)	1 (25.0)	0 (0.0)	5 (7.8)	0 (0.0)	0 (0.0)
Renal disease^d^	6 (5.3)	1 (4.0)	1 (12.5)	0 (0.0)	0 (0.0)	0 (0.0)	2 (3.1)	1 (6.3)	2 (22.2)

Abbreviations: CaN, calcineurin; i, inhibitors; IL, interleukin; PASI, Psoriasis Area and Severity Index; PDE4, phosphodiesterase 4; SD, standard deviation; TNF, tumor necrosis factor; Vit A deriv, vitamin A derivatives.

^a^ From the date of diagnosis of psoriasis to the start of follow‐up.

^b^ The duration of psoriasis was calculated with *n* = 113 for all, and *n* = 8 for those receiving vitamin A derivatives.

^c^ At the start of follow‐up.

^d^ Duplicate counts.

### 3.2 Proportion of treatment changes and drug persistence

Of 114 patients who started systemic drugs during the study, 62 patients (54.4%) switched to a second systemic drug. As a second systemic drug, IL‐17, PDE4, and IL‐23 inhibitors were commonly administrated (25.8%, 24.2%, and 17.7%, respectively) (Table 2). Following the second systemic drug, 28 patients (24.6%) switched to a third systemic drug, and the tendency of selecting a third systemic drug was almost similar to that of selecting the second, although the percentage of calcineurin inhibitors selected as a third systemic drug increased slightly. Furthermore, the fourth systemic drugs were administered to 16 patients (14.0%). Treatment changes of all patients who started systemic drugs during the study are shown in swimmer plots in Supporting Information Figure S1a–g.

**TABLE 2:** Proportion of treatment changes and subsequent systemic drugs.

	Order of administration						
	First	Second	Third	Fourth	Fifth	Sixth	Seventh
No. of patients (%)	114 (100)	62 (54.4)	28 (24.6)	16 (14.0)	7 (6.1)	3 (2.6)	1 (0.9)
Biologic	25 (21.9)	33 (53.2)	13 (46.4)	9 (56.3)	4 (57.1)	1 (33.3)	0 (0.0)
TNFi	8 (7.0)	4 (6.5)	3 (10.7)	3 (18.8)	0 (0.0)	1 (33.3)	0 (0.0)
IL‐12/23i	2 (1.8)	2 (3.2)	0 (0.0)	1 (6.3)	0 (0.0)	0 (0.0)	0 (0.0)
IL‐23i	4 (3.5)	11 (17.7)	4 (14.3)	2 (12.5)	3 (42.9)	0 (0.0)	0 (0.0)
IL‐17i	11 (9.6)	16 (25.8)	6 (21.4)	3 (18.8)	1 (14.3)	0 (0.0)	0 (0.0)
PDE4i	64 (56.1)	15 (24.2)	5 (17.9)	5 (31.3)	0 (0.0)	1 (33.3)	1 (100.0)
CaNi	16 (14.0)	8 (12.9)	6 (21.4)	2 (12.5)	3 (42.9)	1 (33.3)	0 (0.0)
Vit A deriv	9 (7.9)	6 (9.7)	4 (14.3)	0 (0.0)	0 (0.0)	0 (0.0)	0 (0.0)

Abbreviations: CaN, calcineurin; i, inhibitors; IL, interleukin; PDE4, phosphodiesterase 4; TNF, tumor necrosis factor; Vit A deriv, vitamin A derivatives.

**FIGURE 1:**
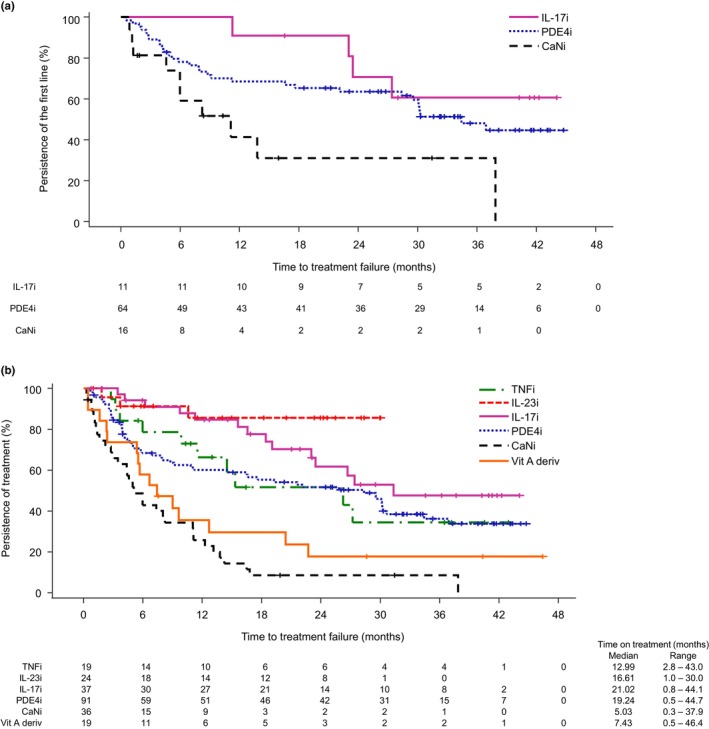
Persistence of systemic treatment (drug survival). Kaplan–Meier survival analysis to analyze curves for drug survival persistence analysis. (a) Persistence of initial systemic drugs started during the study (in more than 10 patients) and (b) persistence of all systemic drugs followed up during the study period (in more than 10 patients). The number of patients at risk for each systemic drug is shown below the time point of the *x* axis. CaN, calcineurin; i, inhibitors; IL, interleukin; PDE4, phosphodiesterase 4; TNF, tumor necrosis factor; Vit A deriv, vitamin A derivatives.

Among the initial systemic drugs started during the study (used by more than 10 patients), the persistence of IL‐17 inhibitors was the longest; 90.9% of patients continued to use them for 18 months (Figure 1a). The persistence of PDE4 inhibitors decreased to 68.5% at 12 months and then decreased gradually to 59.5% at 30 months. The persistence of calcineurin inhibitors decreased to 41.4% at 12 months.

Among all systemic drugs followed up during the study period, IL‐23 and IL‐17 inhibitors had the longest 12‐month persistence (85.6% and 84.7%, respectively) (Figure 1b). The persistence of TNF and PDE4 inhibitors decreased to 66.3% and 60.1%, respectively, in 12 months. The 12‐month persistence of vitamin A derivatives and calcineurin inhibitors was relatively short at 35.5% and 25.8%, respectively (Figure 1b).

### 3.3 Patterns of treatment changes and the reasons

Among 114 patients with PsO, there were 209 treatment changes during the study period (Table 3), and the incidence of treatment change was 59.1/100 patient‐years. Of 209 treatment changes, 132 cases were changes in patients receiving monotherapy; the most common treatment change from monotherapy was interruption in 71 cases (53.8%), followed by switching to another monotherapy in 39 cases (29.5%) and adding another drug in 22 cases (multidrug therapy, 16.7%). Among patients who changed treatment from PDE4 inhibitors (*n* = 57), there were 33 interruptions (57.9%), 16 switched to monotherapy (28.1%), and eight added another drug (multidrug therapy, 14.0%). Among the patients who changed treatment from calcineurin inhibitors (*n* = 32), 13 interrupted treatment (40.6%), 10 switched to another monotherapy (31.3%), and nine added another drug (multidrug therapy, 28.1%). Of 71 cases of interruptions, 55 cases restarted monotherapy; following interruption, an IL‐17 or PDE4 inhibitor was restarted in 15 cases (27.3%) each. Supporting Information Table S2 presents a breakdown of the combinations used in 22 cases of multidrug therapy.

**TABLE 3:** Patterns of treatment change.

Before treatment change			After treatment change								
No. of treatment changes (%)			Monotherapy							Multidrug therapy^a^	Interruption
Monotherapy	Total	132	39 (29.5)							22 (16.7)	71 (53.8)
			TNFi	IL‐12/23i	IL‐23i	IL‐17i	PDE4i	CaNi	Vit A deriv		
	TNFi	10	1 (10.0)	0 (0.0)	1 (10.0)	0 (0.0)	1 (10.0)	0 (0.0)	0 (0.0)	1 (10.0)	6 (60.0)
	IL‐12/23i	4	0 (0.0)	0 (0.0)	3 (75.0)	0 (0.0)	0 (0.0)	0 (0.0)	0 (0.0)	1 (25.0)	0 (0.0)
	IL‐23i	2	1 (50.0)	0 (0.0)	0 (0.0)	0 (0.0)	0 (0.0)	0 (0.0)	0 (0.0)	0 (0.0)	1 (50.0)
	IL‐17i	14	1 (7.1)	0 (0.0)	0 (0.0)	1 (7.1)	0 (0.0)	0 (0.0)	0 (0.0)	2 (14.3)	10 (71.4)
	PDE4i	57	1 (1.8)	0 (0.0)	4 (7.0)	5 (8.8)	0 (0.0)	5 (8.8)	1 (1.8)	8 (14.0)	33 (57.9)
	CaNi	32	0 (0.0)	0 (0.0)	3 (9.4)	4 (12.5)	3 (9.4)	0 (0.0)	0 (0.0)	9 (28.1)	13 (40.6)
	Vit A deriv	13	0 (0.0)	0 (0.0)	0 (0.0)	0 (0.0)	4 (30.8)	0 (0.0)	0 (0.0)	1 (7.7)	8 (61.5)
Multidrug therapy^a^		22	21 (95.5)							1 (4.5)	0 (0.0)
			2 (9.1)	3 (13.6)	5 (22.7)	0 (0.0)	7 (31.8)	3 (13.6)	1 (4.5)		
Interruption		55	55 (100.0)							0 (0.0)	0 (0.0)
			5 (9.1)	0 (0.0)	3 (5.5)	15 (27.3)	15 (27.3)	11 (20.0)	6 (10.9)		
Total treatment change		209									

Abbreviations: CaN, calcineurin; i, inhibitors; IL, interleukin; PDE4, phosphodiesterase 4; TNF, tumor necrosis factor; Vit A deriv, vitamin A derivatives.

^a^ Multidrug therapy due to the addition of another drug.

Of 132 treatment changes from monotherapy, the most common reason for treatment change was lack of efficacy in 45 cases (34.1%), followed by AE in 24 cases (18.2%) (Table 4). In 22 cases (16.7%), other drugs were added to the PsO treatment, although we could not determine the reasons. The most common reason for treatment change from oral drugs was lack of efficacy (vitamin A derivatives 46.2%, calcineurin inhibitors 37.5%, and PDE4 inhibitors 35.1%), and the addition of another drug was also frequently observed for oral drugs (calcineurin inhibitors 28.1%, PDE4 inhibitors 14.0%, and vitamin A derivatives 7.7%). As for PDE4 inhibitors, of 20 cases of treatment changes due to lack of efficacy, nine switched to biologics (TNF, IL‐23, or IL‐17 inhibitors) and six switched to oral drugs (calcineurin inhibitors or vitamin A derivatives). Treatment changes caused by AEs occurred more frequently in TNF and PDE4 inhibitors (30.0% and 24.6%, respectively), and most of the treatment changes caused by AEs were interruptions.

**TABLE 4:** Reasons for treatment changes from monotherapy and subsequent drug.

Treatment change cases (*n* ≥ 10) no. of cases (%)	Reason for treatment changes						
	Total	Occurrence of a new type of psoriasis	AE	Lack of efficacy	Addition of other drugs	Sufficient efficacy	Other
Treatment changes from monotherapy	132 (100.0)	1 (0.8)	24 (18.2)	45 (34.1)	22 (16.7)	8 (6.1)	32 (24.2)
**Treatment changes from TNFi**	**10 (100.0)**	**—**	**3 (30.0)**	**1 (10.0)**	**1 (10.0)**	**—**	**5 (50.0)**
Treatment after TNFi							
TNFi	1 (10.0)		1 (10.0)				
IL‐12/23i							
IL‐23i	1 (10.0)						1 (10.0)
IL‐17i							
PDE4i	1 (10.0)			1 (10.0)			
CaNi							
Vit A deriv							
Multidrug therapy	1 (10.0)				1 (10.0)		
Interruption	6 (60.0)		2 (20.0)				4 (40.0)
**Treatment changes from IL‐17i**	**14 (100.0)**	**1 (7.1)**	**2 (14.3)**	**2 (14.3)**	**2 (14.3)**	**2 (14.3)**	**5 (35.7)**
Treatment after IL‐17i							
TNFi	1 (7.1)			1 (7.1)			
IL‐12/23i							
IL‐23i							
IL‐17i	1 (7.1)			1 (7.1)			
PDE4i							
CaNi							
Vit A deriv							
Multidrug therapy	2 (14.3)				2 (14.3)		
Interruption	10 (71.4)	1 (7.1)	2 (14.3)			2 (14.3)	5 (35.7)
**Treatment changes from PDE4i**	**57 (100.0)**	**—**	**14 (24.6)**	**20 (35.1)**	**8 (14.0)**	**4 (7.0)**	**11 (19.3)**
Treatment after PDE4i							
TNFi	1 (1.8)			1 (1.8)			
IL‐12/23i							
IL‐23i	4 (7.0)			3 (5.3)		1 (1.8)	
IL‐17i	5 (8.8)			5 (8.8)			
PDE4i							
CaNi	5 (8.8)			5 (8.8)			
Vit A deriv	1 (1.8)			1 (1.8)			
Multidrug therapy	8 (14.0)				8 (14.0)		
Interruption	33 (57.9)		14 (24.6)	5 (8.8)		3 (5.3)	11 (19.3)
**Treatment changes from CaNi**	**32 (100.0)**	**—**	**3 (9.4)**	**12 (37.5)**	**9 (28.1)**	**1 (3.1)**	**7 (21.9)**
Treatment after CaNi							
TNFi							
IL‐12/23i							
IL‐23i	3 (9.4)			3 (9.4)			
IL‐17i	4 (12.5)			4 (12.5)			
PDE4i	3 (9.4)		1 (3.1)	2 (6.3)			
CaNi							
Vit A deriv							
Multidrug therapy	9 (28.1)				9 (28.1)		
Interruption	13 (40.6)		2 (6.3)	3 (9.4)		1 (3.1)	7 (21.9)
**Treatment changes from Vit A deriv**	**13 (100.0)**	**—**	**1 (7.7)**	**6 (46.2)**	**1 (7.7)**	**1 (7.7)**	**4 (30.8)**
Treatment after Vit A deriv							
TNFi							
IL‐12/23i							
IL‐23i							
IL‐17i							
PDE4i	4 (30.8)			4 (30.8)			
CaNi							
Vit A deriv							
Multidrug therapy	1 (7.7)				1 (7.7)		
Interruption	8 (61.5)		1 (7.7)	2 (15.4)		1 (7.7)	4 (30.8)

Abbreviations: AE, adverse event; CaN, calcineurin; i, inhibitors; IL, interleukin; n, number of cases; PDE4, phosphodiesterase 4; TNF, tumor necrosis factor; Vit A deriv, vitamin A derivatives.

**FIGURE 2:**
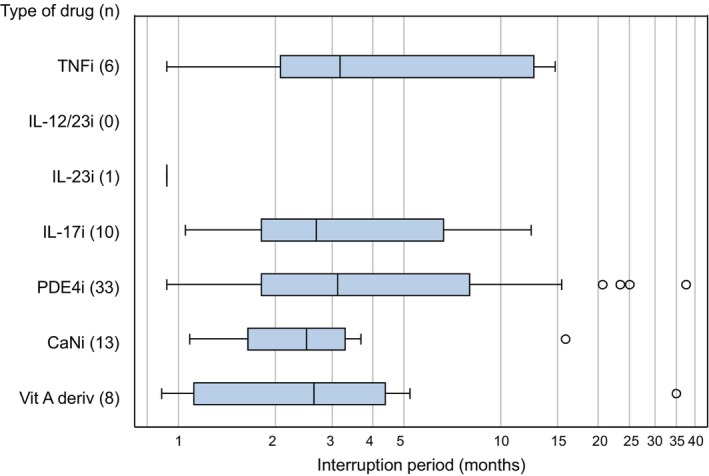
Durations of interruption after the ceasing of systemic drugs. Within each box, vertical black lines represent median values. Boxed extended from the first quartile (Q1) to third quartile (Q3) of data distribution of interruption period for each systemic drug. Lower whiskers represent minimum observation values above the lower fence [Q1 − 1.5 times interquartile range (IQR)], and upper whiskers represent maximum observation values above upper fence (Q3 + 1.5 times IQR). Dots denote outliers. CaN, calcineurin; i, inhibitors; IL, interleukin; PDE4, phosphodiesterase 4; TNF, tumor necrosis factor; Vit A deriv, vitamin A derivatives.

The median interruption period was approximately 3 months (Figure 2). To determine the extent to which the total number of interruptions and the reasons varied by the duration of the interruption period, when the interruption period was defined as ≥28, ≥60, ≥90, and ≥180 days after ceasing treatment with systemic drugs, the total number of interruptions and the reasons were counted (Table 5). Overall, the total number of interruptions decreased gradually as the definition of interruption period was extended.

**TABLE 5:** Number of interruptions, their reasons, and duration.

Interruption period (days)	Treatment changes from monotherapy *n* = 132	Number of interruptions per reason (%)					Total number of interruptions
		Occurrence of a new type of psoriasis	Adverse event	Lack of efficacy	Sufficient efficacy	Other	
≥28		1 (0.8)	22 (16.7)	10 (7.6)	7 (5.3)	31 (23.5)	71 (53.8)
≥60		1 (0.8)	13 (9.8)	6 (4.5)	6 (4.5)	22 (16.7)	48 (36.4)
≥90		0 (0.0)	8 (6.1)	4 (3.0)	5 (3.8)	16 (12.1)	33 (25.0)
≥180		0 (0.0)	7 (5.3)	3 (2.3)	2 (1.5)	5 (3.8)	17 (12.9)

Abbreviation: *n*, number of treatment change cases.

### 3.4 Concomitant use of phototherapy

Of 114 patients, 23 patients (20.2%) used concomitant phototherapy: 22 (19.3%) used UVB and one (0.9%) used PUVA. Concomitant use of phototherapy was observed in 29.7% and 26.3% of patients treated with PDE4 inhibitors and vitamin A derivatives, respectively. In contrast, patients receiving biologics and calcineurin inhibitors did not receive concomitant phototherapy (Table 6).

**TABLE 6:** Concomitant use of phototherapy.

	No. of patients	Phototherapy, *n* (%)
Patients, *n* (%)	114	23 (20.2)
UVB		22 (19.3)
PUVA		1 (0.9)
Concomitant use of phototherapy^a^		
TNFi	19	0 (0.0)
IL‐12/23i	5	0 (0.0)
IL‐23i	24	0 (0.0)
IL‐17i	37	0 (0.0)
PDE4i	91	27 (29.7)
CaNi	36	0 (0.0)
Vit A deriv	19	5 (26.3)

Abbreviations: CaN, calcineurin; i, inhibitors; IL, interleukin; PDE4, phosphodiesterase 4; PUVA, psoralen ultraviolet; TNF, tumor necrosis factor; UVB, ultraviolet B; Vit A deriv, vitamin A derivatives.

^a^ Cases that were duplicated were considered. When multidrug therapy was combined with phototherapy, the number of times phototherapy was used concurrently with each drug was counted.

## 4 DISCUSSION

### 4.1 Study population and the initial systemic drug

This study aimed to understand real‐world, long‐term PsO treatment patterns, reasons for treatment changes, and relationships between the reasons for treatment change and subsequent treatment. We retrospectively reviewed the medical charts of 114 patients with PsO who started systemic treatment at four Japanese medical institutes between January 2017 and December 2020. We followed their systemic treatment courses, including up to the seven drug changes, during a mean follow‐up period of 37.2 months.

This study clarified that Japanese patients with PsO started a systemic drug after a mean period of 7.8 years following the first PsO diagnosis. In contrast, a retrospective database research study in the USA reported that patients with PsO started systemic treatment within 2 years of PsO diagnosis.^26^ Although there are many differences in the healthcare system between Japan and the USA, this may be because of limited access to JDA‐certified medical institutions in charge of prescribing biologics to patients with PsO. As of April 2023, 870 JDA‐certified medical institutions have dispensed biologics to patients with PsO in Japan; however, these institutions were particularly insufficient in rural areas.^27^ Improving access to biologics for PsO can lead to the early provision of appropriate systemic treatment for Japanese patients, particularly those unsatisfied with existing topical and conventional systemic treatments.

Apremilast is a PDE4 inhibitor approved in Japan in December 2016 for treating adult patients with moderate‐to‐severe PsO. The percentage of Japanese patients with PsO who were treated with PDE4 inhibitors increased dramatically during the period between 2016 and 2018, which partially overlapped with the study period.^28^ Our study also demonstrated that PDE4 inhibitors were used most frequently (56.1%) in patients who started a systemic drug between 2017 and 2020. In contrast, 21.9% of patients started with biologics during the study, nearly twice the proportion of patients treated with biologics in a previous study using the Japan Medical Data Center (JMDC) database.^20^ However, the proportion of patients undergoing treatment with PDE4 inhibitors in this and the JMDC study was comparable. This study was conducted in JDA‐certified medical institutions, where many patients with moderate‐to‐severe PsO seek medical care and are prescribed biologics, which may explain the present results. The JDA guidance^17^ states that biologics should be reserved for (1) patients with skin lesions extending over ≥10% of the body surface and who have not responded adequately to existing systemic therapies, including phototherapy, or (2) patients with hard‐to‐treat skin lesions or joint symptoms that persist despite existing treatments and whose quality of life is severely impaired. The mean PASI score of patients who started biologics during the study was higher than that of patients starting PDE4 inhibitors during the study. This indicates that patients with more severe diseases tend to start systemic treatment with biologics, as specified in the JDA guidance.^17^

Although we focused on how comorbidities can impact systemic drug selection, we did not observe any clear trend between comorbidities and initial systemic drugs started during the study.

### 4.2 Proportion of treatment changes and drug persistence

More than half of the patients who started a systemic drug during the study switched to another systemic drug. Approximately half of the patients who switched to a second systemic drug switched to a third systemic drug, and roughly half of them switched to a fourth systemic drug. Similarly, high frequencies of treatment changes were reported in a previous publication.^20^ Furthermore, these findings may reflect the actual clinical practice in Japan, where patients with moderate‐to‐severe PsO have difficulty receiving appropriate systemic drugs. Of the patients who switched to second or subsequent systemic drugs, approximately half selected biologics, and approximately one‐fourth selected PDE4 inhibitors. Thus, biologics and PDE4 inhibitors were commonly used as drug alternatives among patients unsatisfied with their ongoing systemic drugs.

In this study, oral drugs tended to have shorter persistence than biologics such as IL‐17 and IL‐23 inhibitors, although PDE4 inhibitors showed relatively longer persistence in oral drugs. Japanese patients with PsO prefer oral to injectable drugs as systemic treatment,^29^ and clinical challenges remain for long‐term treatment with oral drugs. The drug persistence of biologics and oral drugs was comparable with that reported in our previous 1‐year retrospective study.^20^ The treatment duration for each oral systemic drug in this study extended approximately two‐ to three‐fold that of our previous study,^20^ probably because of the extended follow‐up period in this study. This study clarified the real‐world, long‐term systemic treatment courses and the tendency of treatment changes in Japanese patients with PsO.

### 4.3 Patterns of treatment changes and the reasons

Interruption of systemic drugs was observed frequently, consistent with a previous retrospective database study.^19^ However, the proportion of interruptions in this study was lower than that in the previous study.^19^ As the definition of interruption period was extended to ≥60, ≥90, or ≥180 days after ceasing systemic drugs, the number of total interruptions gradually decreased. Many patients who interrupted systemic drugs tended to restart any systemic treatment within 6 months after interruption, regardless of the reasons. Other reasons may also include poor drug adherence or financial reasons. In such cases, the majority of patients restarted any systemic drug within 3–6 months.

Patients with PsO experience significant treatment burdens, including healthcare costs, time costs, and physical burdens (e.g., injection frequency).^30^, ^31^ In particular, the cost of biologics may discourage patients from starting treatment with biologics as an initial treatment. However, frequent treatment changes, including the use of biologics, can lead to an increase in healthcare costs comparable to the costs of long‐term use of biologics.^32^ Moreover, improper treatment interruption can lead to the exacerbation of PsO and increased risk of comorbidities, including psoriatic arthritis. Providing continuous and appropriate treatment to patients with chronic PsO is crucial. Lack of efficacy was the most common reason for switching from monotherapy, followed by AEs. Although the reasons for adding other drugs were not analyzed in this study, it is plausible to assume that it was due to the inefficacy of the original drug. This suggests that approximately half of the treatment changes from monotherapy were due to a lack of efficacy. Treatment changes from oral drugs were more frequent than those from biologics, and lack of efficacy was a more common reason for oral drugs than biologics. These results suggest that there is still room for improvement in the appropriate use of systemic drugs for PsO in Japan.

### 4.4 Concomitant use of phototherapy

Phototherapy was performed sporadically during the study period and was used as a supportive treatment for PsO. A recent study showed that phototherapy combined with a PDE4 inhibitor could enhance treatment efficacy without impacting safety.^33^ Our results may reflect the acceptance of phototherapy combined with a PDE4 inhibitor as one of the treatment options in Japan.

### 4.5 Limitations

This study had several limitations. First, data regarding the PASI scores were limited owing to the retrospective study design, and we could not assess detailed patterns of treatment changes by PsO severity. Second, the clinical outcomes for each patient who underwent various treatment changes could not be evaluated because of limited information regarding the severity and disease condition at the end of the follow‐up. Third, some biologics—in particular anti‐IL‐23p19 agents—were approved in Japan during the study period, such as guselkumab (March 2018), risankizumab (March 2019), certolizumab pegol (December 2019), and tildrakizumab (June 2020), therefore the proportion of patients treated with these agents might be underestimated. Fourth, because the number of patients receiving each agent was limited, it was not possible to analyze the effect of patient background on persistence with each agent. Finally, given the small sample size and that the four participating institutes were tertiary institutions, the patient population in this study may not fully reflect that of general medical institutions in Japan. Nevertheless, the institutions were representative tertiary hospitals of medical universities in Japan, and play an important role in community medicine in each region of the country. Medical chart reviews can be the best source for understanding the current state of disease treatment and clinical outcomes. This retrospective chart review provides real‐world data regarding the systemic treatment of PsO in Japanese patients in a hospital setting.

## 5 CONCLUSION

Japanese patients with moderate‐to‐severe PsO started systemic treatment after a mean period of 7.8 years following the first diagnosis, reflecting the insufficient availability of systemic treatment for patients with chronic PsO in Japan. Most patients started with oral drugs as their first option, but in many cases these drugs were less effective, leading to more frequent changes in treatment. Our study suggests that it is desirable to have oral drugs with longer‐lasting, higher efficacy for moderate‐to‐severe cases of psoriasis. Biologics were used in a limited number of patients with relatively higher severity of PsO and patients who interrupted oral drugs due to lack of efficacy. Biologics had relatively higher persistence than oral drugs, but a certain number of patients treated with biologics changed treatment because of AEs or other reasons. As more systemic treatment options become available for PsO treatment, using each drug properly will be even more crucial. We believe that our research has shed light on each existing drug's usage patterns, contributing to the optimization of future psoriasis treatment.

## CONFLICT OF INTEREST STATEMENT

This clinical trial was sponsored by Bristol Myers Squibb. Y.T. has received honoraria and/or grants from AbbVie, Boehringer Ingelheim, Bristol‐Myers Squibb KK, Celgene, Eisai, Eli Lilly, Janssen, Kyowa Kirin, LEO Pharma, Maruho, Meiji Seika Pharma, Mitsubishi Tanabe Pharma, Novartis Pharma, Sun Pharmaceutical Industries Limited, Taiho Pharmaceutical, Torii Pharmaceutical, and UCB Pharma. M.K. has received grants or honoraria from Mebix, Leo Pharma, Boehringer Ingelheim, Bristol Myers Squibb, Taiho Yakuhin, Kyowa Kirin, Abbvie, Torii Yakuhin, Tanabe Mitsubishi, Eisai, Sun Pharma, and Maruho. Y.O. has received research grants from Sun Pharma Japan, Eisai, Torii, Maruho, AbbVie, Taiho, Kyowa Kirin, and JIMRO and honoraria from AbbVie, Eli Lilly, Janssen Pharma, Kyowa Kirin, Maruho, Novartis Pharma, Taiho, UCB Pharma, Boehringer Ingelheim, Bristol Myers Squibb, CMIC, Eli Lilly, EPS, LEO Pharma, Mediscience Planning, Otsuka, Pfizer, and UCB Pharma. K.H. and K.T. are employees of and stockholders in Bristol Myers Squibb. A.M. has received research grants, consultancy fees, and/or speaker's fees from AbbVie, Amgen, Boehringer‐Ingelheim, Bristol‐Myers Squibb, Eisai, Eli Lilly Japan, Janssen Pharmaceutical, Kyowa Kirin, LEO Pharma, Maruho, Minophagen Pharmaceutical, Mitsubishi Tanabe Pharma, Nippon Kayaku, Novartis, Pfizer Japan, Sun Pharma Japan, Taiho Pharmaceutical, Torii Pharmaceutical, UCB Japan, and Ushio.

## Supporting information


Figure S1.

Table S1.

## Data Availability

The Bristol Myers Squibb policy on data sharing may be found at https://www.bms.com/researchers‐and‐partners/clinical‐trials‐and‐research/disclosure‐commitment.html.

## References

[1] Rajguru JP , Maya D , Kumar D , Suri P , Bhardwaj S , Patel ND . Update on psoriasis: a review. J Family Med Prim Care. 2020;9:20–24. 32110559 10.4103/jfmpc.jfmpc_689_19 PMC7014874

[2] Kubota K , Kamijima Y , Sato T , Ooba N , Koide D , Iizuka H , et al. Epidemiology of psoriasis and palmoplantar pustulosis: a nationwide study using the Japanese national claims database. BMJ Open. 2015;5:e006450. 10.1136/bmjopen-2014-006450 PMC4298108 25588781

[3] Kouris A , Platsidaki E , Kouskoukis C , Christodoulou C . Psychological parameters of psoriasis. Psychiatriki. 2017;28:54–59. 28541239 10.22365/jpsych.2017.281.54

[4] Mustonen A , Mattila K , Leino M , Koulu L , Tuominen R . How much of the productivity losses among psoriasis patients are due to psoriasis. BMC Health Serv Res. 2015;15:87. 25888995 10.1186/s12913-015-0752-0 PMC4352284

[5] Bonanad C , González‐Parra E , Rivera R , Carrascosa JM , Daudén E , Olveira A , et al. Clinical, diagnostic, and therapeutic implications in psoriasis associated with cardiovascular disease. Actas Dermosifiliogr. 2017;108:800–808. 28610662 10.1016/j.ad.2016.12.023

[6] Gelfand JM , Neimann AL , Shin DB , Wang X , Margolis DJ , Troxel AB . Risk of myocardial infarction in patients with psoriasis. JAMA. 2006;296:1735–1741. 17032986 10.1001/jama.296.14.1735

[7] Samarasekera EJ , Neilson JM , Warren RB , Parnham J , Smith CH . Incidence of cardiovascular disease in individuals with psoriasis: a systematic review and meta‐analysis. J Invest Dermatol. 2013;133:2340–2346. 23528816 10.1038/jid.2013.149

[8] Gyldenløve M , Storgaard H , Holst JJ , Vilsbøll T , Knop FK , Skov L . Patients with psoriasis are insulin resistant. J Am Acad Dermatol. 2015;72:599–605. 25653028 10.1016/j.jaad.2015.01.004

[9] Danielsen K , Wilsgaard T , Olsen AO , Eggen AE , Olsen K , Cassano PA , et al. Elevated odds of metabolic syndrome in psoriasis: a population‐based study of age and sex differences. Br J Dermatol. 2015;172:419–427. 25059341 10.1111/bjd.13288 PMC4338759

[10] Psoriasis YF . Comorbidities. J Dermatol. 2021;48:732–740. 33763899 10.1111/1346-8138.15840 PMC8252780

[11] Karmacharya P , Chakradhar R , Ogdie A . The epidemiology of psoriatic arthritis: a literature review. Best Pract Res Clin Rheumatol. 2021;35:101692. 34016528 10.1016/j.berh.2021.101692

[12] Singla S , Putman M , Liew J , Gordon K . Association between biological immunotherapy for psoriasis and time to incident inflammatory arthritis: a retrospective cohort study. Lancet Rheumatol. 2023;5:e200–e207. 38251522 10.1016/S2665-9913(23)00034-6

[13] Karamata VV , Gandhi AM , Patel PP , Sutaria A , Desai MK . A study of the use of drugs in patients suffering from psoriasis and their impact on quality of life. Indian J Pharm. 2017;49:84–88. 10.4103/ijp.IJP_166_16 PMC5351244 28458428

[14] Reich K , Korge B , Magnolo N , Manasterski M , Schwichtenberg U , Staubach‐Renz P , et al. Quality‐of‐life outcomes, effectiveness and tolerability of apremilast in patients with plaque psoriasis and routine German dermatology care: results from LAPIS‐PSO. Dermatol Ther (Heidelb). 2022;12:203–221. 34913153 10.1007/s13555-021-00658-x PMC8776950

[15] Lacour JP , Bewley A , Hammond E , Hansen JB , Horne L , Paul C , et al. Association between patient‐ and physician‐reported outcomes in patients with moderate‐to‐severe plaque psoriasis treated with biologics in real life (PSO‐BIO‐REAL). Dermatol Ther (Heidelb). 2020;10:1099–1109. 32761560 10.1007/s13555-020-00428-1 PMC7477065

[16] Iizuka H . Pyramid plan for psiasis treatment [in Japanese] visual dermatology, Tokyo. Japan: Gakken Inc.; 2017.

[17] Saeki H , Terui T , Morita A , Sano S , Imafuku S , Asahina A , et al. Japanese guidance for use of biologics for psoriasis (the 2019 version). J Dermatol. 2020;47:201–222. 31916326 10.1111/1346-8138.15196

[18] Saeki H , Mabuchi T , Asahina A , Abe M , Igarashi A , Imafuku S , et al. English version of Japanese guidance for the use of oral Janus kinase inhibitors (JAK1 and TYK2 inhibitors) in the treatments of psoriasis. J Dermatol. 2023;50:e138–e150. 37132187 10.1111/1346-8138.16797

[19] Wu B , Muser E , Teeple A , Pericone CD , Feldman SR . Treatment adherence and persistence of five commonly prescribed medications for moderate to severe psoriasis in a US commercially insured population. J Dermatolog Treat. 2021;32:595–602. 31714168 10.1080/09546634.2019.1687828

[20] Tada Y , Kim H , Spanopoulos D , Habiro K , Tsuritani K , Yamada Y , et al. Treatment patterns, healthcare resource utilization, and costs in patients with moderate‐to‐severe psoriasis treated with systemic therapy in Japan: a retrospective claims database study. J Dermatol. 2022;49:1106–1117. 35946343 10.1111/1346-8138.16543 PMC9804179

[21] Thai S , Zhuo J , Zhong Y , Xia Q , Chen X , Bao Y , et al. Real‐world treatment patterns and healthcare costs in patients with psoriasis taking systemic oral or biologic therapies. J Dermatolog Treat. 2023;34:2176708. 36794863 10.1080/09546634.2023.2176708

[22] Armstrong AW , Gooderham M , Warren RB , Papp KA , Strober B , Thaçi D , et al. Deucravacitinib versus placebo and apremilast in moderate to severe plaque psoriasis: efficacy and safety results from the 52‐week, randomized, double‐blinded, placebo‐controlled phase 3 POETYK PSO‐1 trial. J Am Acad Dermatol. 2023;88:29–39. 35820547 10.1016/j.jaad.2022.07.002

[23] Imafuku S , Tada Y , Hippeli L , Banerjee S , Morita A , Ohtsuki M . Efficacy and safety of the selective TYK2 inhibitor, deucravacitinib, in Japanese patients with moderate to severe plaque psoriasis: subgroup analysis of a randomized, double‐blind, placebo‐controlled, global phase 3 trial. J Dermatol. 2023;50:588–595. 36882942 10.1111/1346-8138.16740

[24] Smith JA , Wehausen B , Richardson I , Zhao Y , Li Y , Herrera V , et al. Treatment changes in patients with moderate to severe psoriasis: a retrospective chart review. J Cutan Med Surg. 2018;22:25–30. 28789566 10.1177/1203475417724438

[25] Carlin CS , Feldman SR , Krueger JG , Menter A , Krueger GG . A 50% reduction in the psoriasis area and severity index (PASI 50) is a clinically significant endpoint in the assessment of psoriasis. J Am Acad Dermatol. 2004;50:859–866. 15153885 10.1016/j.jaad.2003.09.014

[26] Murage MJ , Kern DM , Chang L , Sonawane K , Malatestinic WN , Quimbo RA , et al. Treatment patterns among patients with psoriasis using a large national payer database in the United States: a retrospective study. J Med Econ. 2018;22:1–9. 30358465 10.1080/13696998.2018.1540424

[27] Japanese Dermatological Association . Medical institutions approved for use of molecular target drug for psoriasis [in Japanese]. [Internet]. 2023 Apr [cited 2023 Jun 1]. Available from: https://www.dermatol.or.jp/modules/biologics/index.php?content_id=4

[28] Kamiya K , Oiso N , Kawada A , Ohtsuki M . Epidemiological survey of the psoriasis patients in the Japanese Society for Psoriasis Research from 2013 to 2018. J Dermatol. 2021;48:864–875. 33580908 10.1111/1346-8138.15803 PMC8247979

[29] Komine M , Kim H , Yi J , Zhong Y , Sakai Y , Crawford B , et al. A discrete choice experiment on oral and injection treatment preferences among moderate‐to‐severe psoriasis patients in Japan. J Dermatol. 2023;50:766–777. 36808765 10.1111/1346-8138.16746

[30] Tada Y , Ishii K , Kimura J , Hanada K , Kawaguchi I . Patient preference for biologic treatments of psoriasis in Japan. J Dermatol. 2019;46:466–477. 30985030 10.1111/1346-8138.14870 PMC6594072

[31] Mustonen A , Mattila K , Leino M , Koulu L , Tuominen R . Psoriasis causes significant economic burden to patients. Dermatol Ther (Heidelb). 2014;4:115–124. 24865468 10.1007/s13555-014-0053-2 PMC4065269

[32] Sruamsiri R , Iwasaki K , Tang W , Mahlich J . Persistence rates and medical costs of biological therapies for psoriasis treatment in Japan: a real‐world data study using a claims database. BMC Dermatol. 2018;18:5. 29996929 10.1186/s12895-018-0074-0 PMC6042444

[33] Morita A , Yamaguchi Y , Tateishi C , Ikumi K , Yamamoto A , Nishihara H , et al. Efficacy and safety of apremilast and phototherapy versus phototherapy only in psoriasis vulgaris. J Dermatol. 2022;49:1211–1220. 36151864 10.1111/1346-8138.16566 PMC10087908

