# Supplementary material for: Treatment patterns of systemic drug use in Japanese patients with plaque psoriasis: A retrospective chart review
Source: J Dermatol. 2023 Nov 30;51(2):210–22. doi: 10.1111/1346-8138.17038 (PMC11484147; doi:10.1111/1346-8138.17038)
Supplement: Supplementary file 1 — Figure S1. [file JDE-51--s002.pptx]

## Slide 1
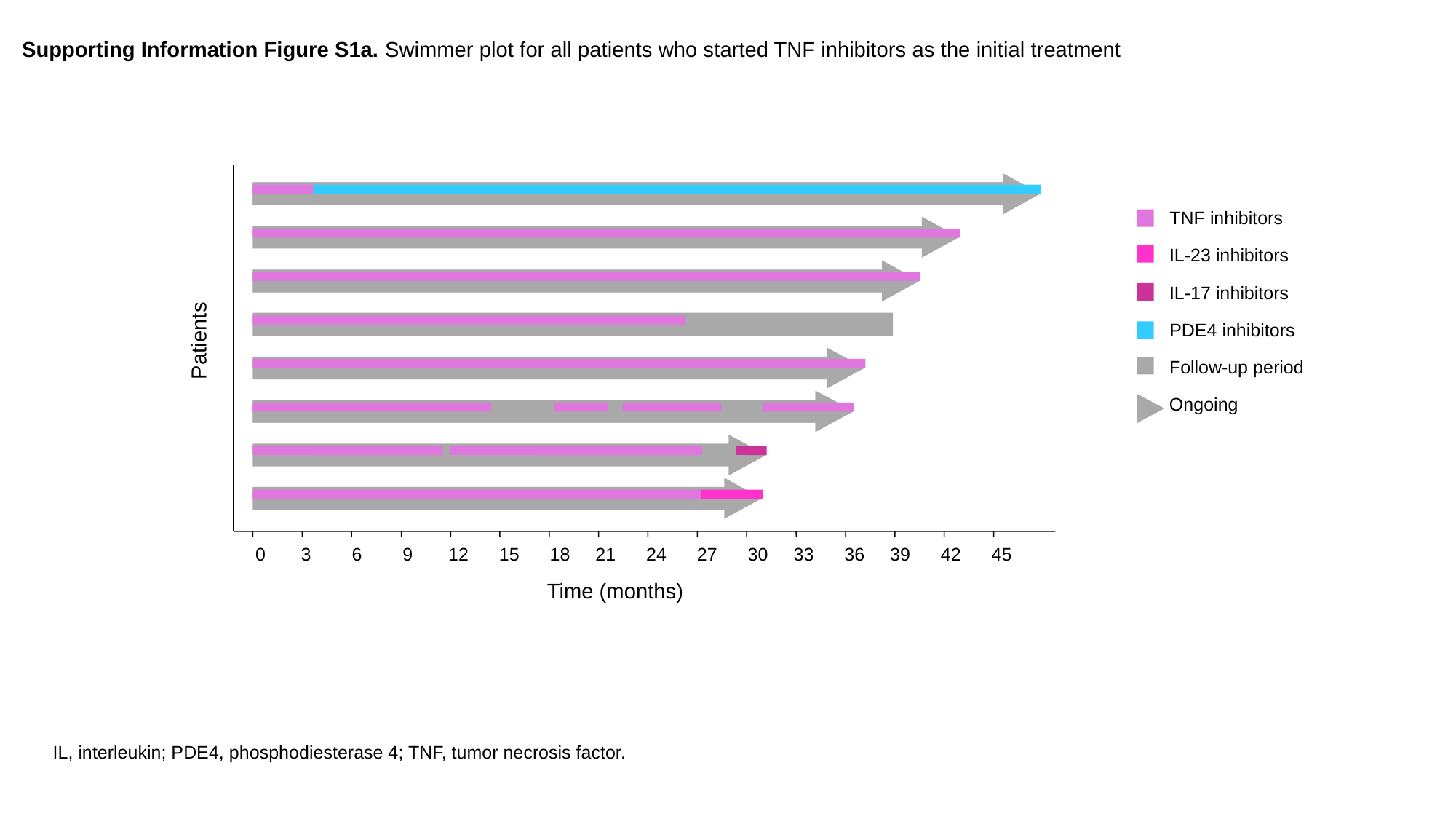

Supporting Information Figure S1a. Swimmer plot for all patients who started TNF inhibitors as the initial treatment
Patients
0 3 6 9 12 15 18 21 24 27 30 33 36 39 42 45
Time (months)
TNF inhibitors
IL-23 inhibitors
IL-17 inhibitors
PDE4 inhibitors
Follow-up period
Ongoing
TNF阻害薬
IL, interleukin; PDE4, phosphodiesterase 4; TNF, tumor necrosis factor.

## Slide 2
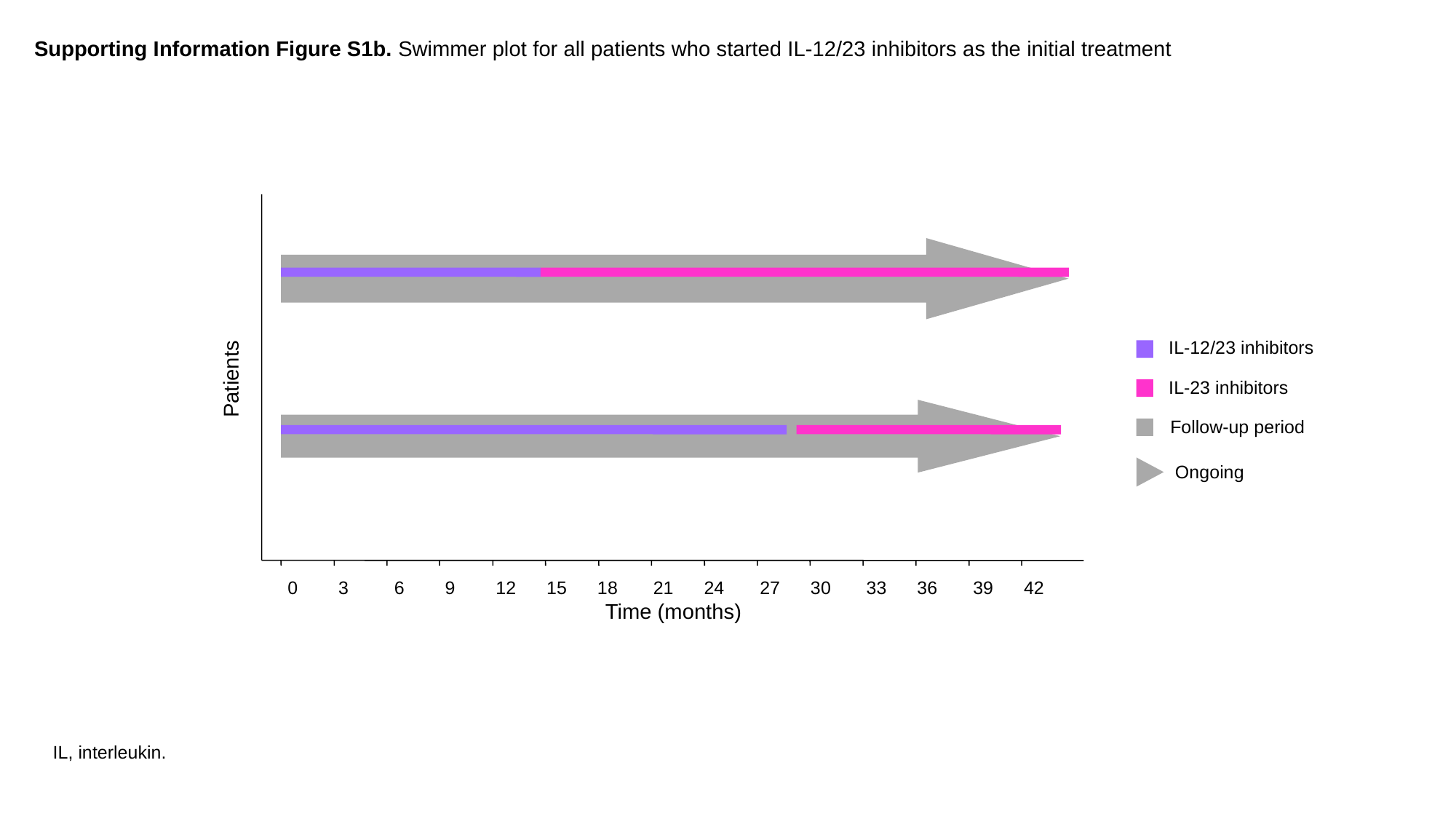

Supporting Information Figure S1b. Swimmer plot for all patients who started IL-12/23 inhibitors as the initial treatment
IL-12/23 inhibitors
ts
n
e
Patients
i
t
IL-23 inhibitors
a
P
Follow-up period
Ongoing
0 3 6 9 12 15 18 21 24 27 30 33 36 39 42
Time (months)
T
i
m
e
(
mo
n
t
h
s
)
IL, interleukin.

## Slide 3
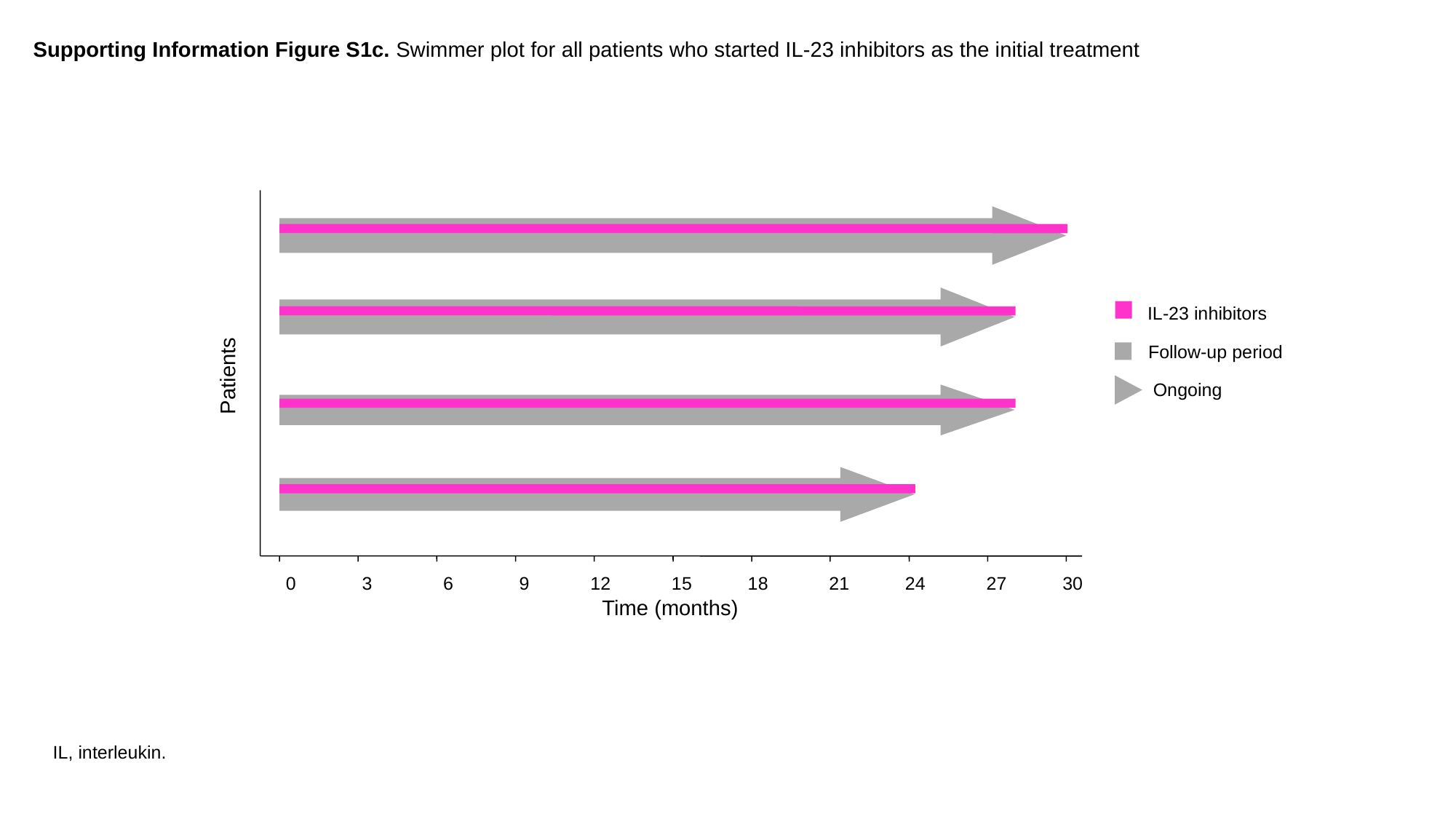

Supporting Information Figure S1c. Swimmer plot for all patients who started IL-23 inhibitors as the initial treatment
IL-23 inhibitors
ts
Follow-up period
n
e
Patients
i
t
Ongoing
a
P
0 3 6 9 12 15 18 21 24 27 30
Time (months)
T
i
m
e
(
mo
n
t
h
s
)
IL, interleukin.

## Slide 4
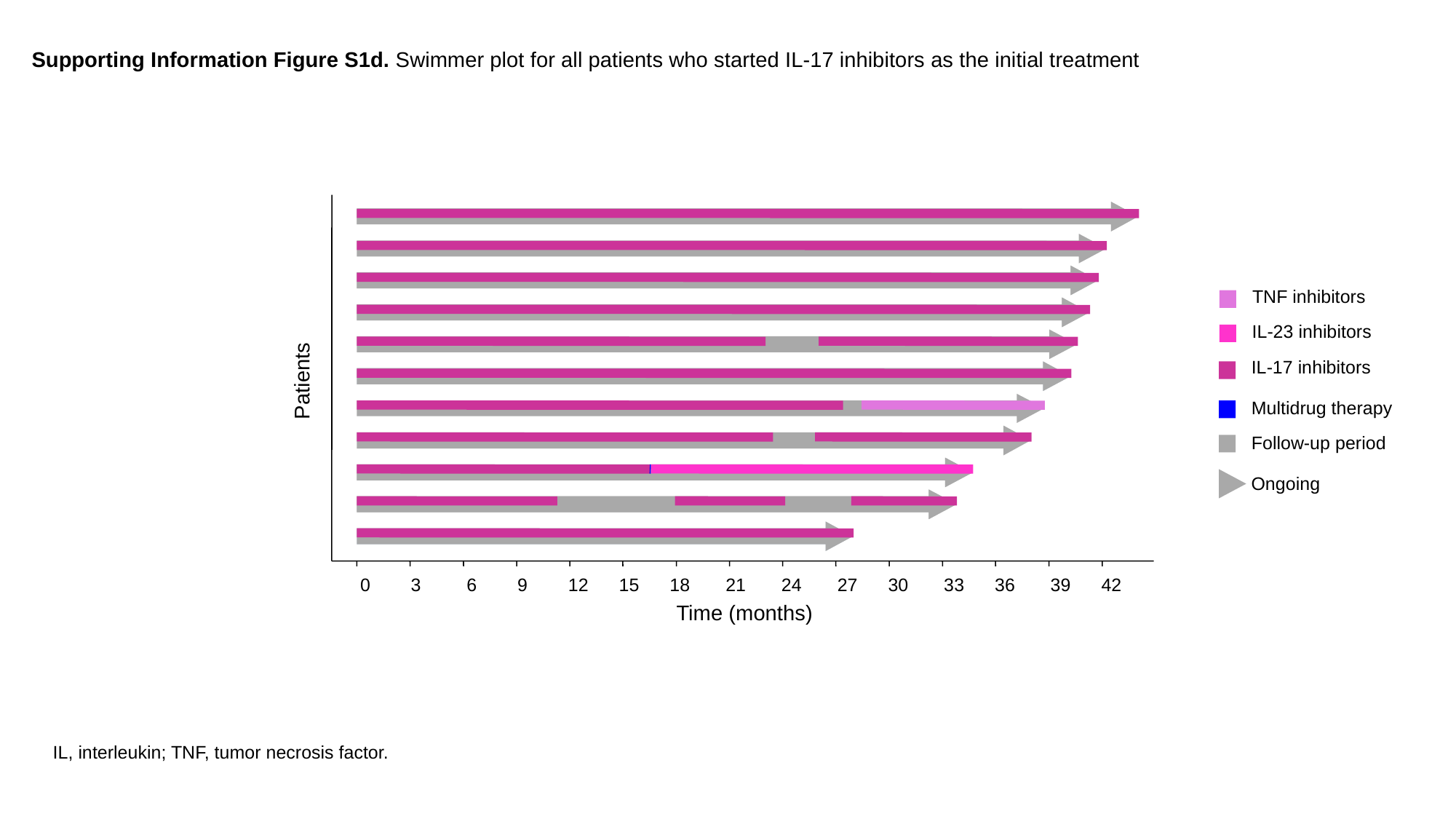

Supporting Information Figure S1d. Swimmer plot for all patients who started IL-17 inhibitors as the initial treatment
ts
n
e
Patients
i
t
a
P
0 3 6 9 12 15 18 21 24 27 30 33 36 39 42
Time (months)
T
i
m
e
(
mo
n
t
h
s
)
TNF inhibitors
IL-23 inhibitors
IL-17 inhibitors
Multidrug therapy
Follow-up period
Ongoing
IL, interleukin; TNF, tumor necrosis factor.

## Slide 5
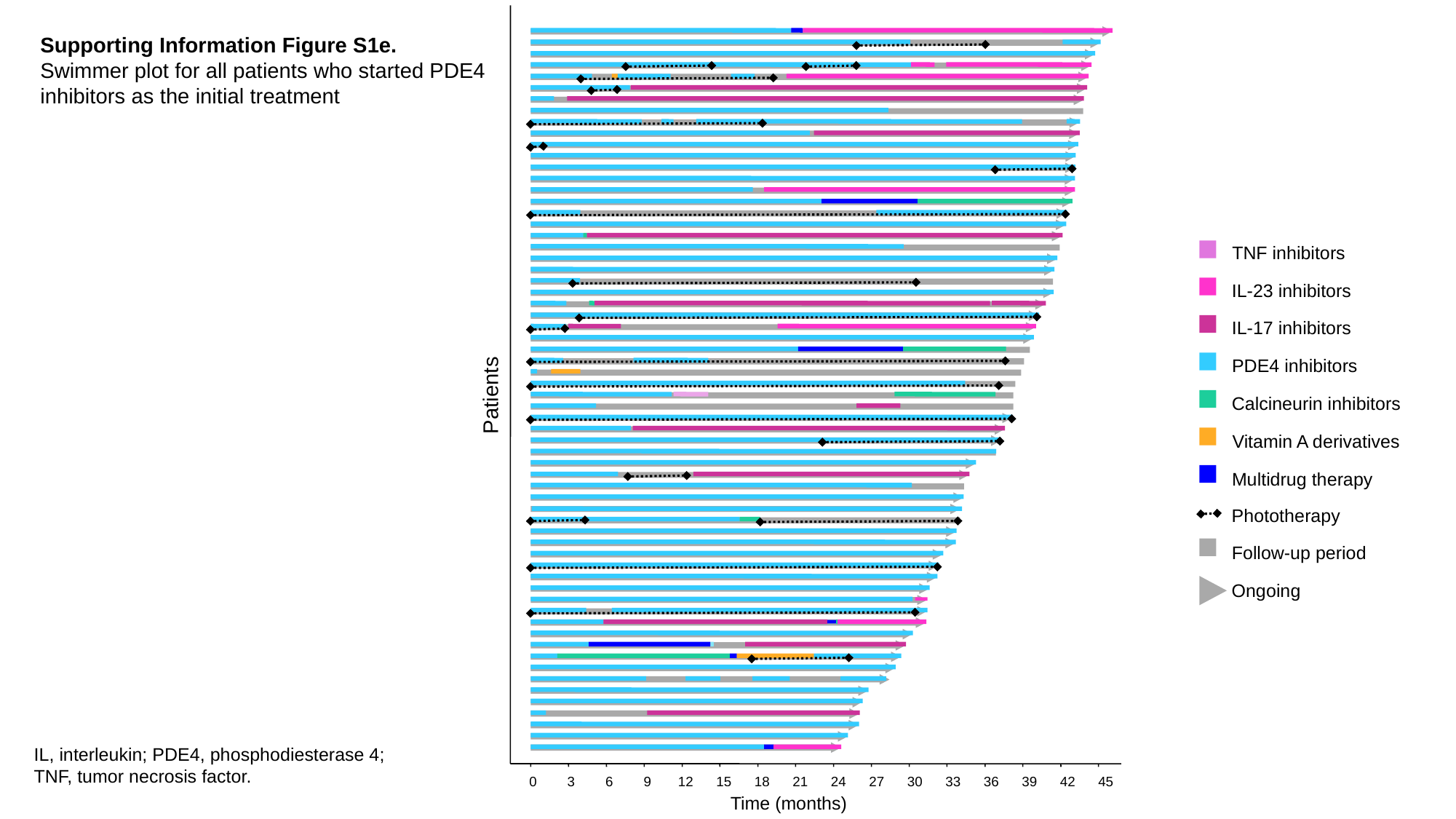

s
t
n
e
Patients
ti
a
P
0 3 6 9 12 15 18 21 24 27 30 33 36 39 42 45
0
3
6
9
1
2
1
5
1
8
2
1
2
4
2
7
3
0
3
3
3
6
3
9
4
2
4
5
T
i
m
e
(
mo
n
t
h
s
)
Time (months)
Supporting Information Figure S1e. Swimmer plot for all patients who started PDE4 inhibitors as the initial treatment
TNF inhibitors
IL-23 inhibitors
IL-17 inhibitors
PDE4 inhibitors
Calcineurin inhibitors
Vitamin A derivatives
Multidrug therapy
Phototherapy
Follow-up period
Ongoing
IL, interleukin; PDE4, phosphodiesterase 4; TNF, tumor necrosis factor.

## Slide 6
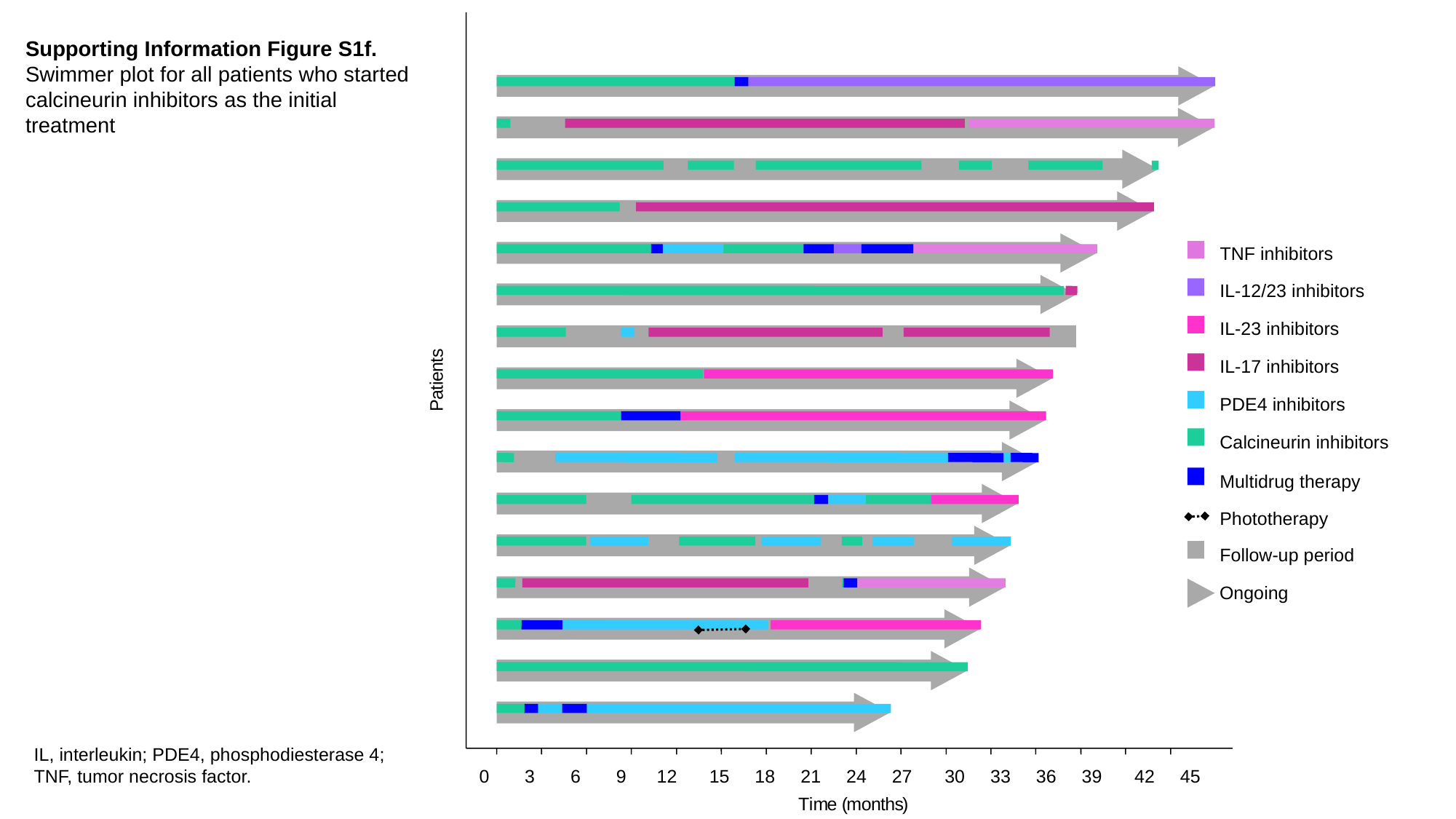

ts
n
e
i
t
a
P
0 3 6 9 12　 15 18 21 24 27　 30 33 36 39 　42 45
0
3
6
9
1
2
1
5
1
8
2
1
2
4
2
7
3
0
3
3
3
6
3
9
4
2
4
5
T
i
m
e
(
mo
n
t
h
s
)
Supporting Information Figure S1f. Swimmer plot for all patients who started calcineurin inhibitors as the initial treatment
TNF inhibitors
IL-12/23 inhibitors
IL-23 inhibitors
IL-17 inhibitors
PDE4 inhibitors
Calcineurin inhibitors
Multidrug therapy
Phototherapy
Follow-up period
Ongoing
IL, interleukin; PDE4, phosphodiesterase 4; TNF, tumor necrosis factor.

## Slide 7
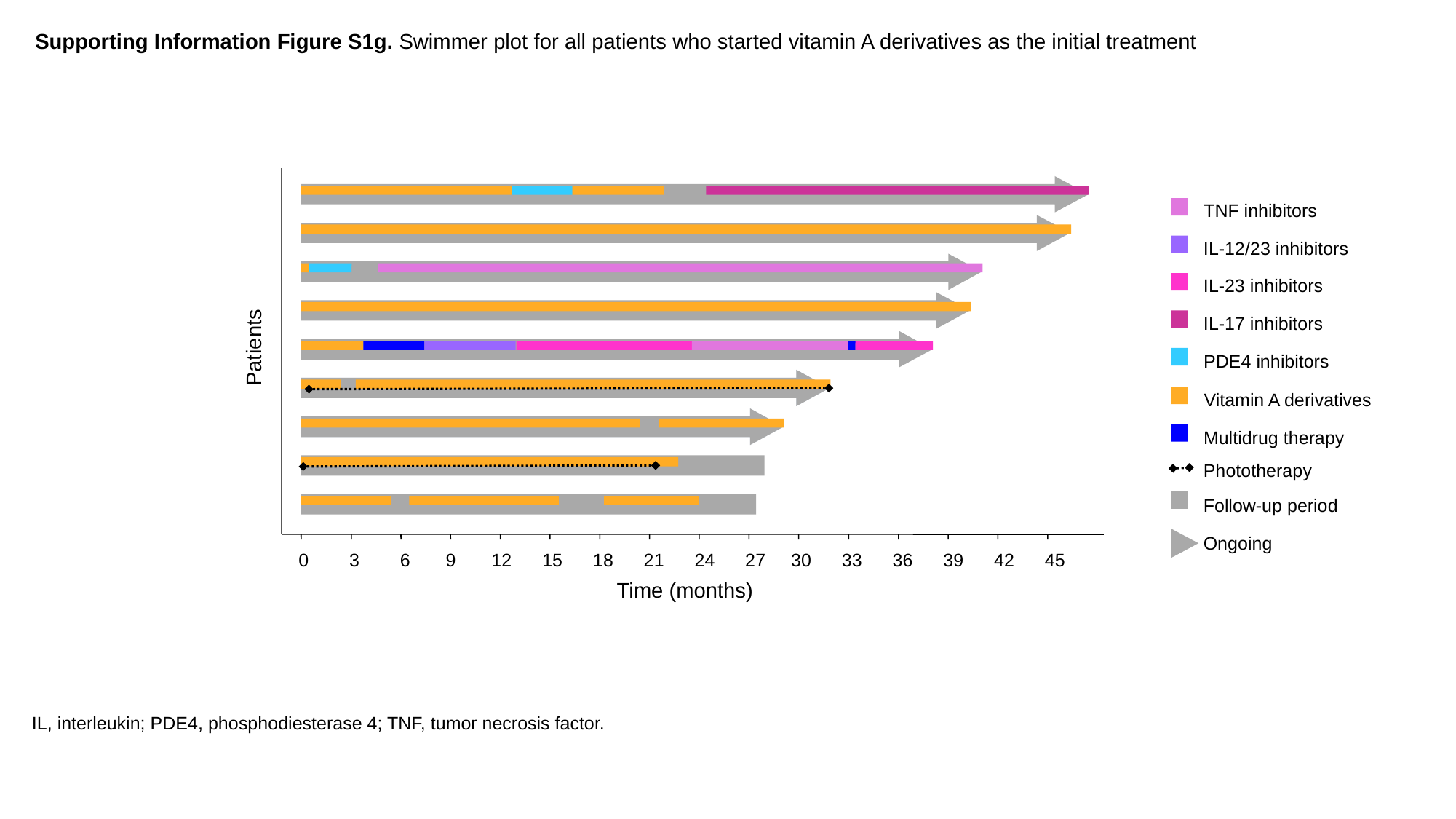

Supporting Information Figure S1g. Swimmer plot for all patients who started vitamin A derivatives as the initial treatment
ts
n
Patients
e
i
t
a
P
0 3 6 9 12 15 18 21 24 27 30 33 36 39 42 45
Time (months)
T
i
m
e
(
mo
n
t
h
s
)
TNF inhibitors
IL-12/23 inhibitors
IL-23 inhibitors
IL-17 inhibitors
PDE4 inhibitors
Vitamin A derivatives
Multidrug therapy
Phototherapy
Follow-up period
Ongoing
IL, interleukin; PDE4, phosphodiesterase 4; TNF, tumor necrosis factor.
