# Supplementary material for: Treatment patterns of systemic drug use in Japanese patients with plaque psoriasis: A retrospective chart review
Source: J Dermatol. 2023 Nov 30;51(2):210–22. doi: 10.1111/1346-8138.17038 (PMC11484147; doi:10.1111/1346-8138.17038)
Supplement: Supplementary file 2 — Table S1. [file JDE-51--s001.docx]

# **Supporting Information Table S1**. Comorbidities associated with systemic treatments started during the study

| **N (%)** | **Total** | **Biologic** | **TNFi** | **IL-12/23i** | **IL-23i** | **IL-17i** | **PDE4i** | **CaNi** | **Vit A deriv** |
| --- | --- | --- | --- | --- | --- | --- | --- | --- | --- |
| No. of patients | 114 | 25 | 8 | 2 | 4 | 11 | 64 | 16 | 9 |
| Comorbidity, Yes | 79 (69.3) | 15 (60.0) | 6 (75.0) | 2 (100.0) | 1 (25.0) | 6 (54.5) | 48 (75.0) | 7 (43.8) | 9 (100.0) |
| Dyslipidemia | 23 (20.2) | 3 (12.0) | 1 (12.5) |  | 1 (25.0) | 1 (9.1) | 16 (25.0) | 1 (6.3) | 3 (33.3) |
| Hypertension | 22 (19.3) | 4 (16.0) | 2 (25.0) | 1 (50.0) |  | 1 (9.1) | 15 (23.4) |  | 3 (33.3) |
| Diabetes mellitus (with chronic comorbidities) | 18 (15.8) | 3 (12.0) | 2 (25.0) |  |  | 1 (9.1) | 12 (18.8) | 1 (6.3) | 2 (22.2) |
| Mild liver disease | 12 (10.5) | 3 (12.0) | 1 (12.5) |  | 1 (25.0) | 1 (9.1) | 8 (12.5) | 1 (6.3) |  |
| Hay fever | 9 (7.9) | 1 (4.0) |  |  |  | 1 (9.1) | 3 (4.7) | 3 (18.8) | 2 (22.2) |
| Hyperuricemia | 8 (7.0) | 3 (12.0) | 1 (12.5) | 1 (50.0) | 1 (25.0) |  | 5 (7.8) |  |  |
| Renal disease | 6 (5.3) | 1 (4.0) | 1 (12.5) |  |  |  | 2 (3.1) | 1 (6.3) | 2 (22.2) |
| Depression | 5 (4.4) | 1 (4.0) |  |  | 1 (25.0) |  | 1 (1.6) |  | 3 (33.3) |
| Rheumatic diseases | 5 (4.4) | 1 (4.0) |  | 1 (50.0) |  |  | 4 (6.3) |  |  |
| Asthma | 4 (3.5) | 1 (4.0) | 1 (12.5) |  |  |  | 1 (1.6) | 1 (6.3) | 1 (11.1) |
| Atopic dermatitis | 4 (3.5) | 1 (4.0) | 1 (12.5) |  |  |  | 2 (3.1) | 1 (6.3) |  |
| Cataracts | 4 (3.5) | 1 (4.0) |  | 1 (50.0) |  |  | 2 (3.1) |  | 1 (11.1) |
| Malignancies including leukemia and lymphoma | 4 (3.5) |  |  |  |  |  | 3 (4.7) |  | 1 (11.1) |
| Hashimoto's disease | 3 (2.6) | 1 (4.0) | 1 (12.5) |  |  |  | 2 (3.1) |  |  |
| Tinea unguium | 3 (2.6) | 1 (4.0) |  |  |  | 1 (9.1) |  |  | 2 (22.2) |
| Constipation | 2 (1.8) |  |  |  |  |  | 2 (3.1) |  |  |
| Hernia | 2 (1.8) |  |  |  |  |  | 1 (1.6) | 1 (6.3) |  |
| Ischemic heart disease, etc. | 2 (1.8) |  |  |  |  |  | 2 (3.1) |  |  |
| Osteoarthritis of the knee | 2 (1.8) |  |  |  |  |  | 1 (1.6) | 1 (6.3) |  |
| Osteoporosis | 2 (1.8) |  |  |  |  |  | 2 (3.1) |  |  |
| Reflux esophagitis | 2 (1.8) |  |  |  |  |  | 1 (1.6) | 1 (6.3) |  |
| Acquired immunodeficiency syndrome | 1 (0.9) |  |  |  |  |  |  |  | 1 (11.1) |
| Allergic rhinitis | 1 (0.9) | 1 (4.0) |  |  |  | 1 (9.1) |  |  |  |
| Androgenetic alopecia | 1 (0.9) |  |  |  |  |  | 1 (1.6) |  |  |
| Anterior thoracic keloids | 1 (0.9) |  |  |  |  |  | 1 (1.6) |  |  |
| Arrhythmia | 1 (0.9) |  |  |  |  |  | 1 (1.6) |  |  |
| Aspirin Asthma | 1 (0.9) |  |  |  |  |  | 1 (1.6) |  |  |
| Asymptomatic stroke | 1 (0.9) |  |  |  |  |  | 1 (1.6) |  |  |
| Atrial fibrillation | 1 (0.9) |  |  |  |  |  | 1 (1.6) |  |  |
| Atrial septal defect | 1 (0.9) |  |  |  |  |  | 1 (1.6) |  |  |
| Attention deficit hyperactivity disorder | 1 (0.9) |  |  |  |  |  |  |  | 1 (11.1) |
| Bipolar affective disorder | 1 (0.9) |  |  |  |  |  | 1 (1.6) |  |  |
| Disc herniation | 1 (0.9) |  |  |  |  |  | 1 (1.6) |  |  |
| Epilepsy | 1 (0.9) |  |  |  |  |  |  |  | 1 (11.1) |
| Facioscapulohumeral muscular dystrophy | 1 (0.9) | 1 (4.0) | 1 (12.5) |  |  |  |  |  |  |
| Glaucoma | 1 (0.9) | 1 (4.0) | 1 (12.5) |  |  |  |  |  |  |
| Hearing loss | 1 (0.9) |  |  |  |  |  | 1 (1.6) |  |  |
| Herpes zoster | 1 (0.9) | 1 (4.0) |  |  |  | 1 (9.1) |  |  |  |
| Hyperneutrophilia | 1 (0.9) |  |  |  |  |  | 1 (1.6) |  |  |
| Hypothyroidism | 1 (0.9) |  |  |  |  |  |  |  | 1 (11.1) |
| Inflammatory bowel disease (UC, CD) | 1 (0.9) | 1 (4.0) |  |  |  | 1 (9.1) |  |  |  |
| Insomnia | 1 (0.9) |  |  |  |  |  | 1 (1.6) |  |  |
| Intervertebral stenosis | 1 (0.9) |  |  |  |  |  | 1 (1.6) |  |  |
| Intraportal thrombosis | 1 (0.9) |  |  |  |  |  | 1 (1.6) |  |  |
| Metal allergy | 1 (0.9) |  |  |  |  |  |  | 1 (6.3) |  |
| Metastatic solid tumors | 1 (0.9) |  |  |  |  |  | 1 (1.6) |  |  |
| Monoclonal immunoglobulinemia | 1 (0.9) |  |  |  |  |  |  |  | 1 (11.1) |
| Ocular hypertension | 1 (0.9) |  |  |  |  |  | 1 (1.6) |  |  |
| Pancytopenia | 1 (0.9) |  |  |  |  |  | 1 (1.6) |  |  |
| Panic disorder | 1 (0.9) |  |  |  |  |  | 1 (1.6) |  |  |
| Prostate enlargement | 1 (0.9) |  |  |  |  |  | 1 (1.6) |  |  |
| Right knee anterior cruciate ligament injury | 1 (0.9) |  |  |  |  |  |  | 1 (6.3) |  |
| Sinusitis | 1 (0.9) | 1 (4.0) | 1 (12.5) |  |  |  |  |  |  |
| Sleep apnea syndrome | 1 (0.9) |  |  |  |  |  | 1 (1.6) |  |  |
| Social anxiety disorder | 1 (0.9) |  |  |  |  |  | 1 (1.6) |  |  |
| Stomach ulcer | 1 (0.9) |  |  |  |  |  |  | 1 (6.3) |  |
| Tinea Nails | 1 (0.9) |  |  |  |  |  |  |  | 1 (11.1) |
| Type II bipolar disorder | 1 (0.9) | 1 (4.0) | 1 (12.5) |  |  |  |  |  |  |
| Unruptured aneurysm | 1 (0.9) | 1 (4.0) |  |  |  | 1 (9.1) |  |  |  |
| Urinary tract stones | 1 (0.9) |  |  |  |  |  | 1 (1.6) |  |  |
| Warts | 1 (0.9) |  |  |  |  |  | 1 (1.6) |  |  |

Abbreviations: CaN, Calcineurin; CD, Crohn's disease; i, inhibitors; IL, interleukin; PDE4, phosphodiesterase 4; TNF, tumor necrosis factor; UC, ulcerative colitis; Vit A deriv, vitamin A derivatives.

# **Supporting Information Table S2.** Patterns of multidrug therapy

| Monotherapy before multidrug therapy | Added drugs | Time to multidrug therapy (months) |
| --- | --- | --- |
| TNFi | IL-23i | 9.4 |
| IL-12/23i | CaNi | 1.8 |
| IL-17i | CaNi | 17.7 |
| IL-17i | IL-23i | 16.5 |
| PDE4i | CaNi | 23.1 |
| PDE4i | CaNi | 21.2 |
| PDE4i | IL-23i | 20.7 |
| PDE4i | IL-23i | 18.5 |
| PDE4i | IL-23i | 14.2 |
| PDE4i | Vit A deriv | 4.6 |
| PDE4i | Vit A deriv | 1.6 |
| PDE4i | IL-17i | 0.5 |
| CaNi | IL-12/23i | 15.9 |
| CaNi | Vit A deriv | 13.7 |
| CaNi | PDE4i | 12.2 |
| CaNi | PDE4i | 10.3 |
| CaNi | IL-23i | 8.3 |
| CaNi | IL-12/23i | 5.4 |
| CaNi | PDE4i | 1.8 |
| CaNi | PDE4i | 1.6 |
| CaNi | TNF-α | 0.1 |
| Vit A deriv | IL-12/23i | 3.8 |

Abbreviations: CaN, Calcineurin; i, inhibitors; IL, interleukin; PDE4, phosphodiesterase 4; TNF, tumor necrosis factor; Vit A deriv, vitamin A derivatives.
